# Antidyslipidemic, Anti-Inflammatory, and Antioxidant Activities of Aqueous Leaf Extract of* Dioscoreophyllum cumminsii* (Stapf) Diels in High-Fat Diet-Fed Rats

**DOI:** 10.1155/2017/8128125

**Published:** 2017-10-23

**Authors:** O. B. Ibitoye, U. M. Ghali, J. B. Adekunle, J. N. Uwazie, T. O. Ajiboye

**Affiliations:** ^1^Antioxidants, Redox Biology and Toxicology Research Laboratory, Department of Biological Sciences, Al-Hikmah University, Ilorin, Nigeria; ^2^Department of Biochemistry, University of Ilorin, Ilorin, Nigeria; ^3^Antioxidants, Redox Biology and Toxicology Research Group, Department of Medical Biochemistry, College of Health Sciences, Nile University of Nigeria, Federal Capital Territory, Nigeria

## Abstract

*Dioscoreophyllum cumminsii* (Stapf) Diels leaves are widely used in the treatment of diabetes, obesity, and cardiovascular related complications in Nigeria. This study investigates the anti-inflammatory and antiobesity effect of aqueous extract of* Dioscoreophyllum cumminsii* leaves in high-fat diet- (HFD-) induced obese rats. HFD-fed rats were given 100, 200, and 400 mgkg^−1^ body weight of aqueous extract of* Dioscoreophyllum cumminsii* leaves for 4 weeks starting from 9th week of HFD treatment.* D. cumminsii* leaves aqueous extract reversed HFD-mediated decrease in the activities of superoxide dismutase, catalase, glutathione peroxidase, glutathione reductase, and glucose 6-phosphate dehydrogenase. Moreover, HFD-mediated elevation in the levels of conjugated dienes, lipid hydroperoxides, malondialdehyde, protein carbonyl, and DNA fragmentation in rats liver was lowered. HFD-mediated alterations in serum total cholesterol, triacylglycerol, high-density lipoprotein cholesterol, low-density lipoprotein cholesterol, and very low-density lipoprotein cholesterol were significantly reversed by the extract. The treatment of HFD-fed rats reduced the levels of insulin, leptin, protein carbonyl, fragmented DNA, and tumour necrosis factor-*α* and interleukin- (IL-) 6 and IL- 8 and increased the adiponectin level. This study showed that aqueous extract of* Dioscoreophyllum cumminsii* leaves has potential antiobesity and anti-inflammatory effects through modulation of obesity-induced inflammation, oxidative stress, and obesity-related disorder in HFD-induced obese rats.

## 1. Introduction

Obesity, excessive visceral accumulation and distribution, is a risk factor in atherosclerosis, cancer, diabetes (type 2), dyslipidemia, and metabolic syndrome [1]. Indeed, its rising prevalence which continues to be a global challenge is associated with high-fat, caloric-dense diets, sedentary life styles, increased urbanization, and psychosocial stress (McLaren, 2007). Adiposity in high-fat consumption has been demonstrated in epidemiological and experimental studies [2, 3] and is associated with adipose tissue inflammation, endoplasmic reticulum stress of adipocyte, and necrosis-like cell death [4]. World Health Organization (WHO) estimated that there are more than 1.1 billion adults overweight in the world and about 115 million individuals suffering from obesity-related problems in low-income and middle-income populations [5]. Drugs including phentermine, fluoxetine, orlistat, sibutramine, and rimonabant are used for the treatment of obesity. However, associated side effects are not limited to nausea, dizziness, insomnia, diarrhea, dyspepsia, and constipation. Thus, there are growing demands for plant derived foods and compounds to manage and treat ailments such as metabolic syndrome, obesity, and diabetes [6]. Indeed, antidiabetic activity and capability of* Dioscoreophyllum cumminsii* leaves have been validated [7, 8]. In furtherance of these, the therapeutic importance of* Dioscoreophyllum cumminsii* leaves in obesity and associated complications was investigated.

*Dioscoreophyllum cumminsii*, a tropical rainforest vine, family Menispermaceae, is known as serendipity berry, (Omu-aja) Yoruba, and Okazi (Igbo) [9]. It is widely distributed in Guinea-Bissau, Sierra Leone, Liberia, Nigeria, Benin, and Congo. Monellin, a content of the fruit, is 3000 times sweeter than sugar [10]. Alkaloids, anthraquinones, cardiac glycosides, flavonoids, phlobatannins, saponins, and tannins are reported phytochemicals in* Dioscoreophyllum cumminsii* leaves [11]. Leaves of this plant are used in the treatment of diarrhea, dysentery, and uterine haemorrhages [11]. Recently, we reported in different models that magnoflorine, jatrorrhizine, and columbamine are responsible for the antidiabetic and protective importance in metabolic syndrome model [7, 8]. As diabetes and metabolic syndrome could result from obesity, we evaluated the effect of aqueous leaf extract of* Dioscoreophyllum cumminsii* on HFD-induced dyslipidemia, inflammation, and oxidative stress.

## 2. Materials and Methods

### 2.1. Experimental Animals

Thirty-five male albino rats* (Rattus norvegicus)* of Wistar strain (141.24 ± 0.32 g) were obtained from the Animal House of Veterinary Physiology, Biochemistry and Pharmacology, University of Ibadan, Nigeria. Rats, kept in clean plastic cages, were placed in well-ventilated house conditions and supplied with feed (Capefeed Ltd., Osogbo, Nigeria) and water ad libitum.

### 2.2. Plant Material and Authentication

*Dioscoreophyllum cumminsii* leaves were collected from Oja Titun, Ilorin, Nigeria. They were authenticated and deposited in the herbarium of Department of Plant Biology, University of Ilorin, Ilorin, Nigeria (UIH 001/1082).

### 2.3. Chemical Reagents and Assay Kits

Disodium salt, hexahydrate and guanidine hydrochloride, ultrapure water, 5,5-dithiobis-2-nitrobenzoic acid (DNTB), and trichloroacetic acid were purchased from Research Organics, 4353 East 49th Street, Cleveland, Ohio 44125; superoxide dismutase, glutathione peroxidase, glutathione reductase, glucose-6-phosphate dehydrogenase, catalase, total cholesterol, triglyceride, and HDL-cholesterol assay kits were purchased from Randox Laboratories Co., Antrim, UK. Adiponectin, insulin, and leptin (enzyme immunoassay kits) were products of Sigma-Aldrich Inc., St. Louis, USA. All other reagents used were products of Sigma-Aldrich Inc., St. Louis, USA.

### 2.4. Preparation of Plant Extract

*Dioscoreophyllum cumminsii* leaves were washed clean with distilled water, air-dried, and pulverized using domestic blender. Pulverized leaves (200 g) were extracted in distilled water (1 L) for 48 h, filtered, and concentrated on water bath. The extract yield (26.60 g) was reconstituted to 100, 200, and 400 mg/kg body weight doses. We reported magnoflorine (1.97 mg/g), jatrorrhizine (1.35 mg/g), and columbamine (2.12 mg/g) as the antidiabetic and antidyslipidemic agents in aqueous leaf extract of* Dioscoreophyllum cumminsii* [7]. The extract was refrigerated all through the experimental period to avoid microbial contamination and maintain its composition.

### 2.5. Feed Composition and Formulation

HFD with composition presented in Table 1 was used for the study and formulated as described by Ajiboye et al. [12].

### 2.6. Animal Grouping and Treatments

Rats (35) were randomized into seven groups (A–G) of five rats each. All rats received HFD for 12 weeks except rats in groups A and C fed with control diet. In addition, rats in groups C–F were gavaged with 400, 100, 200, and 400 mg/kg BW of aqueous extract of* D. cumminsii*, respectively, for 4 weeks starting from 9th week of diet treatments. Group A rats, which served as control, were gavaged with distilled water (1 mL), while group G rats received 400 mg/kg BW metformin [8], reference drug, for 4 weeks starting from 9th week. This study was approved by Al-Hikmah University Ethical Committee on the use of laboratory animals (HUI/ECULA/014/009) and all treatments were done in accordance with the Guidelines of National Research Council's Guide for the Care and Use of Laboratory Animals [13].

### 2.7. Preparation of Serum and Tissue Homogenate

Rats were anaesthetized with diethyl ether and sacrificed 24 h after the last day of the experimental period. Blood collected from the jugular vein was allowed to clot for 15 min and centrifuged for 5 min at 500*g* for serum collection. Liver was excised and homogenized in sucrose-Tris buffer (0.25 mol/L sucrose, 10 mmol/L Tris-HCl, pH 7.4).

### 2.8. Biochemical Assays

#### 2.8.1. Blood Insulin, Adipokines, and Cytokines

Adiponectin, insulin, leptin, tumour necrosis factor-*α*, interleukin-6, and interleukin-8 were determined as described in manufacturer's assay kit manual.

#### 2.8.2. Lipid Profile

Serum TC, TAG, and HDLc were determined as described in commercial kits (Randox Laboratories Ltd., Antrim, UK). LDLc and VLDLc were calculated using the following expression:

(1) LDLc=0.2×TAGLDLc=TC−HDLc+VLDLc.

Cardiac index (CI), atherogenic index, and coronary artery index were estimated as described by Kang et al. [14], Kayamori and Igarashi [15], and Ajiboye et al. [16], respectively.

#### 2.8.3. Antioxidant Enzymes and Oxidative Stress Biomarkers

*Superoxide Dismutase*. Superoxide dismutase in the liver of rats was determined as described by Misra and Fridovich [17]. The assay mixture consisted of liver homogenate (0.2 mL), 2.5 mL carbonate buffer (0.05 M, pH 10.2), and freshly prepared 0.3 mM epinephrine (0.3 mL). Increase in absorbance was monitored at 480 nm every 30 s for 150 s. A unit of enzyme activity was defined as 50% inhibition of the rate of autoxidation of epinephrine as determined by change in absorbance/min at 480 nm.

*Catalase*. Catalase activity was determined as described by Beers and Sizer [18]. The assay mixture consisted of 2 mL phosphate buffer and 30 mM H_2_O_2_ and liver homogenate (50 *μ*L). Absorbance was read at 240 nm for 1 min and the activity was calculated using the extinction coefficient of H_2_O_2_ (43.6 M cm^−1^).

*Glutathione Peroxidase and Glutathione Reductase*. Activities of glutathione peroxide and glutathione reductase were determined as described in commercial kits (Randox Laboratories Ltd., Antrim, UK).

*Reduced Glutathione (GSH)*. Glutathione content of liver was determined as described by Ellman [19]. Briefly, liver homogenate (1.0 mL) was mixed with 0.1 mL of 25% trichloroacetic acid (TCA). The mixture was centrifuged at 5,000 ×g for 10 min to remove precipitate. Supernatant (0.1 mL) was mixed with 2 mL of 0.6 mM DTNB prepared in 0.2 M sodium phosphate buffer pH (8.0). Absorbance was read at 412 nm.

*Lipid Peroxidation Products*. Lipid peroxidation products were determined as described for conjugated dienes [20], lipid hydroxide [20], and malondialdehyde [20].

*Protein Carbonyl and Fragmented DNA*. Protein carbonyl and fragmented DNA contents of the liver were determined as described by Levine et al. [21] and Burton [22], respectively.

### 2.9. Statistical Analysis

All the data were expressed as the mean ± SEM of five replicates unless stated otherwise. Analysis of variance (ANOVA) followed by Tukey-Kramer test for difference between means was used to detect any significant difference between the treatment groups in this study. Statistical evaluation of data was performed with SPSS version 20.0. Differences were considered statistically significant at *p* < 0.05.

## 3. Results

### 3.1. Insulin, Leptin, Adiponectin, and Inflammatory Biomarkers

Serum insulin and leptin of HFD-fed rats increased significantly (*p* < 0.05) by 298.36 and 114.92%, respectively, when compared with the control (Table 2). This increase was significantly lowered by aqueous leaf extract of* Dioscoreophyllum cumminsii* (100, 200, and 400 mg/kg BW). Conversely, HFD-mediated increase in serum adiponectin was significantly attenuated by the extract and compared well with the reference drug (Table 2).

**Figure 1:**
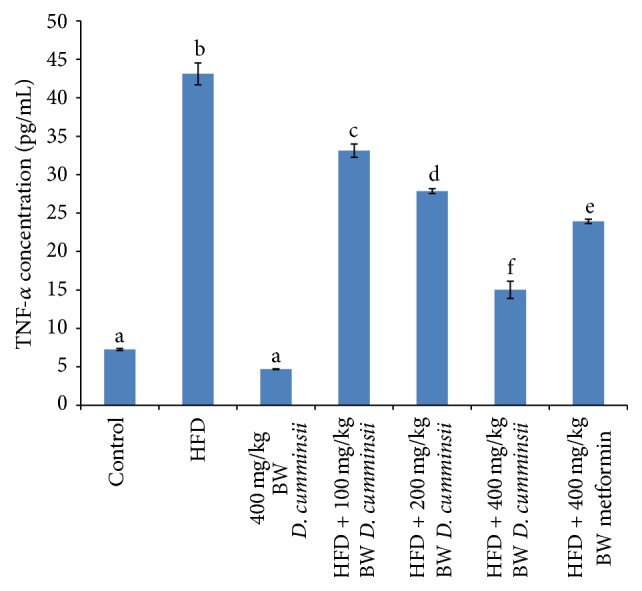
TNF-*α* concentration in the serum of HFD-fed rats following the administration of aqueous* Dioscoreophyllum cumminsii* leaves. Values are mean ± SEM of five determinations and are statistically significant at *p* < 0.05. Bars with different alphabetical superscript are significantly different at *p* < 0.05. HFD: high-fat diet; TNF-*α*: tumour necrosis factor-*α*.

**Figure 2:**
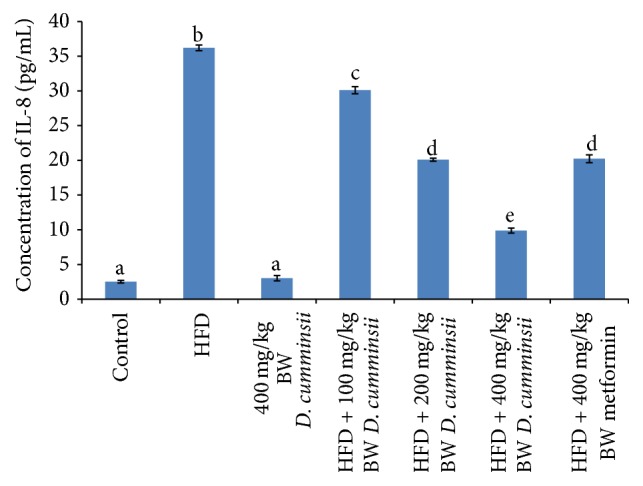
Serum concentration of IL-8 of HFD-fed rats following the administration of aqueous extract of* Dioscoreophyllum cumminsii* leaves. Values are mean ± SEM of five determinations and are statistically significant at *p* < 0.05. Bars with different alphabetical superscript are significantly different at *p* < 0.05. HFD: high-fat diet; IL-8: interleukin-8.

**Figure 3:**
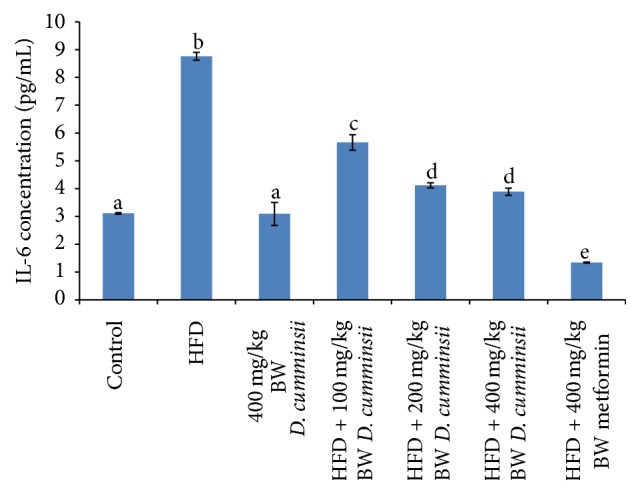
Serum concentration of IL-6 of HFD-fed rats following the administration of aqueous extract of* Dioscoreophyllum cumminsii* leaves. Values are mean ± SEM of five determinations and are statistically significant at *p* < 0.05. Bars with different alphabetical superscript are significantly different at *p* < 0.05. HFD: high-fat diet; IL-6: interleukin-6.

Inflammatory biomarkers, TNF-*α*, IL-6, and IL-8, increased significantly in the serum of HFD-fed rats in comparison with control rats (Figures 1–3). The extract produced dose dependent decrease in these biomarkers and compared well with the reference drug (Figures 1–3). The highest dose (400 mg/kg BW) of* D. cumminsii* leaves produced profound decrease in HFD-mediated increase in serum TNF-*α*, IL-6, and IL-8, respectively.

### 3.2. Lipid Profile

TC, TAG, VLDLc, and LDLc of HFD-fed rats increased significantly with concomitant decreased HDLc in comparison with control rats. Administration of* D. cumminsii* leaves extract significantly reversed HFD-mediated alterations in these parameters. Indeed, the highest dose (400 mg/kg body weight) produced 85.11, 64.04, 51.60, 72.92, and 60.88% reversal of TC, TAG VLDLc, LDLc, and HDLc, respectively, and compared significantly with reference drug, metformin (Table 3).

### 3.3. Antioxidant Enzymes

Activities of antioxidant enzymes, superoxide dismutase, catalase, glutathione peroxidase, glutathione reductase, and glucose 6-phosphate dehydrogenase, in the liver of HFD-fed rats decreased significantly (*p* < 0.05) when compared to control rats. This decrease was significantly reversed by aqueous leaf extract of* D. cumminsii* in dose dependent manner (Table 4), which compared significantly with metformin treated rats.

### 3.4. Oxidative Stress Biomarkers

**Figure 4:**
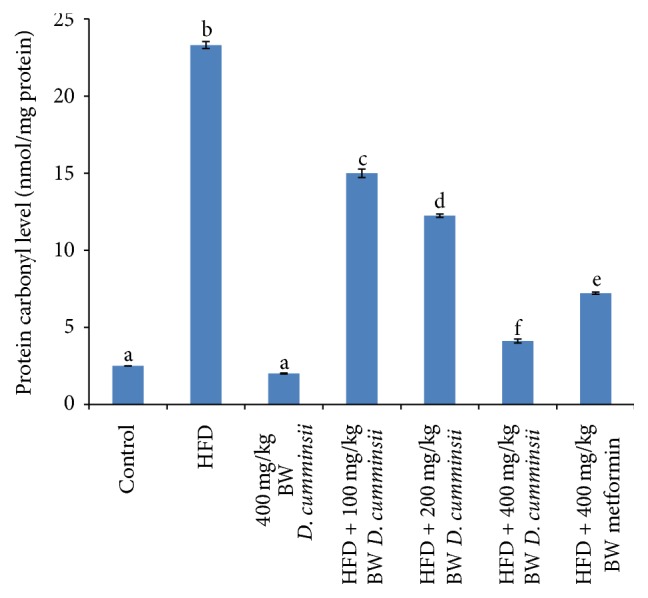
Protein carbonyl level in the liver of HFD-fed rats following the administration of aqueous extract of* Dioscoreophyllum cumminsii* leaves. Values are mean ± SEM of five determinations and are statistically significant at *p* < 0.05. Bars with different alphabetical superscript are significantly different at *p* < 0.05. HFD: high-fat diet.

**Figure 5:**
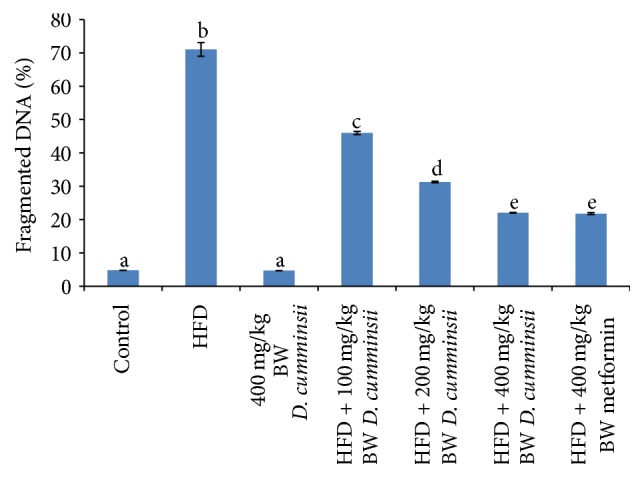
Fragmented DNA (%) in the liver of HFD-fed rats following the administration of* Dioscoreophyllum cumminsii* leaves. Values are mean ± SEM of five determinations and are statistically significant at *p* < 0.05. Bars with different alphabetical superscript are significantly different at *p* < 0.05. HFD: high-fat diet.

Lipid peroxidation products, conjugated dienes, lipid hydroperoxides, and malondialdehyde, in the liver of HFD-fed rats increased significantly by 608.59, 214.82, and 257.61% when compared to control rats. Administration of aqueous leaf extract of* D. cumminsii* significantly lowered HFD-mediated increase in levels of conjugated dienes, malondialdehyde, and lipid hydroperoxides when compared to the control rats (Table 5). Similar reduction was observed for HFD-fed rats treated with metformin. Also, protein carbonyl, product of protein oxidation, and fragmented DNA of HFD-fed rats were lowered by extract administration (Figures 4 and 5).

## 4. Discussion

Demands for health promoting/maintenance foods have led to increase in investigations into the bioactive constituents (phenolic acids, polyphenols, and micro- and macronutrients) conferring the medicinal properties [6]. Although studies have documented the usefulness of* D. cumminsii* leaves in the management of diabetes and high-fructose-induced metabolic syndrome, no study has evaluated the effect on HFD-induced obesity. This study thus presents the antidyslipidemic, anti-inflammatory, and antioxidant activities of aqueous leaf extract of* Dioscoreophyllum cumminsii* (Stapf) Diels in HFD-fed rats.

Leptin, adiponectin, and insulin are indicators of body mass fats and energy imbalance and are present in obesity [23, 24]. The increase in serum leptin and insulin of HFD-fed rats is in consonance with previous studies [25–27]. Reversal of HFD*-*mediated increase in leptin and insulin by the extract suggests inhibition of lipogenesis and stimulation of lipolysis and reduction of intracellular lipid levels in skeletal muscle, liver, and pancreatic *β*-cells, leading to improved insulin sensitivity [24] and decreased lipid accumulation in adipocytes [28].

Previous studies have demonstrated decrease in adiponectin level in HFD-fed rats and have implicated its involvement in diseases presenting obesity [24]. This could be associated with insulin resistance and hyperinsulinemia [29]. The reversal of HFD-mediated decrease in adiponectin by aqueous leaf extract of* Dioscoreophyllum cumminsii* could have resulted from improved insulin sensitivity, as evident in this study, leading to decreased flow of free fatty acids and stimulating glucose utilization and fatty acid oxidation [30]. In addition, this may protect cardiovascular system and reduce incidence of myocardial infarction [29].

Elevated levels of TC, TG, VLDLc, and LDLc with concomitant reduction in HDLc characterize the dyslipidemic changes reported for HFD [12, 31]. Indeed, TC, TG, VLDLc, LDLc, and HDLc indicate disordered lipid metabolism and predisposition to cardiovascular disease [12, 31, 32]. These alterations could predispose the risk of developing atherosclerosis and cardiovascular diseases [33], while reduction in HDL cholesterol could intensify the development of atherosclerosis and cardiovascular diseases. Indeed, studies have demonstrated the importance of aqueous leaf extract of* Dioscoreophyllum cumminsii* in the regulation of dyslipidemia in diabetic and metabolic HFD-fed rats [7, 8]. Thus, the reversal of HFD-fed rats mediated alterations in lipid profile by aqueous leaf extract of* D. cumminsii* suggests antidyslipidemic activity of the extract.

Oxidative stress associated with consumption of HFD results from overwhelmed antioxidant enzymes, which act in concerted manner to detoxify reactive oxygen species [34]. The decreased antioxidant enzymes observed in this study have been documented in HFD-fed rats [12, 32, 35, 36]. Reversal of HFD-mediated decrease in these enzymes suggests antioxidant activity of the extract, although, in a different animal model, antioxidant activities of* D. cumminsii* have been reported [7, 8].

Lipid peroxidation, protein oxidation, and DNA fragmentation are consequential effects of overwhelmed antioxidant defense system. Elevated levels of lipid peroxidation products, CD, LH, and MDA, in this study are in accordance with previous studies [12, 32, 37, 38]. This may lead to disorganization and functional loss of membrane [39]. Similar increased protein carbonyl and fragmented DNA, associated with HFD consumption [40–43], indicate oxidative stressed rats. The capability of* D. cumminsii* to reverse the increase in oxidative stress biomarkers further provided the antioxidant capability of the extract.

## 5. Conclusion

Arising from the data obtained from this study, it is evident from the reversal of HFD-mediated alterations in proinflammatory cytokines, metabolic hormones, and antioxidant enzymes that aqueous leaf extract of* Dioscoreophyllum cumminsii* leaves possesses antioxidants, antidyslipidemic, and anti-inflammatory properties.

## Figures and Tables

**Table 1:** Feed composition and formulation.

Feed component	Control diet (g/kg)	High-fat diet (g/kg)
Corn starch	506	396
Casilan 90^*∗*^	250	250
Lard	40	140
Cholesterol	0	10
Sucrose	100	100
Rice husk	40	40
DL-methionine	4	4
Lysine	10	10
Vitamin mix^*∗∗*^	10	10
Mineral mix^*∗∗∗*^	40	40

^*∗*^Casilan 90 (g 100 g^−1^), energy (1572 kg 100 g^−1^), protein (90 g), carbohydrate (0.3 g), fat (1.0 g), fibre (trace), sodium (0.03 mg), and calcium (1400 mg). ^*∗∗*^Vitamin mix (per kg of diet): thiamine hydrochloride (6 mg), pyridoxine hydrochloride (7 mg), nicotine acid (30 mg), calcium pantothenate (16 mg), folic acid (2 mg), biotin (0.2 mg), Cyanocobalamin (0.01 mg), retinol palmitate (4000 IU), cholecalciferol (100 IU), *α*-tocopherol acetate (50 IU), menadione (0.05 mg), and choline chloride (2 g). ^*∗∗∗*^Mineral mix (g kg^−1^): CoCl_2_·6H_2_O (0.001), CuSO_4_·5H_2_O (0.079), MnSO_4_·7H_2_O (0.178), KI (0.033), NaCl (3.573), ZnCO_3_ (1.60), CaSO_4_ (11.61), MgSO_4_·7H_2_O (2.292), K_2_HPO_4_ (10.559), and FeSO_4_·7H_2_O (1.075).

**Table 2:** Insulin, leptin, and adiponectin levels of HFD-fed rats following oral administration of aqueous leaf extract of *Dioscoreophyllum cumminsii*.

Group	Insulin (IU/L)	Leptin (ng/mL)	Adiponectin (mg/mL)
Control	0.61 ± 0.09^a^	1.81 ± 0.04^b^	42.21 ± 2.10^d^
HFD	2.43 ± 0.12^e^	3.89 ± 0.03^e^	20.65 ± 1.23^a^
400 mg/kg body weight of extract	0.67 ± 0.07^a^	1.65 ± 0.03^a^	41.02 ± 1.62^d^
HFD + 100 mg/kg body weight extract	2.01 ± 0.24^d^	3.04 ± 0.25^d^	23.89 ± 1.05^a^
HFD + 200 mg/kg body weight extract	1.71 ± 0.13^c^	2.82 ± 0.16^c^	28.72 ± 0.96^b^
HFD + 400 mg/kg body weight extract	1.00 ± 0.08^b^	1.83 ± 0.10^b^	36.01 ± 0.58^c^
HFD + 400 mg/kg body weight metformin	1.65 ± 0.11^c^	2.72 ± 0.91^c^	35.98 ± 0.73^c^

Values are mean ± SEM of five determinations and are considered statistically significant at *p* < 0.05. HFD: high-fat diet. Values with different alphabetical superscript are significantly different (*p* < 0.05).

**Table 3:** Lipid profile HFD-fed rats following oral administration of aqueous leaf extract of *Dioscoreophyllum cumminsii*.

Group	Total cholesterol	Triglyceride	VLDL cholesterol	LDL cholesterol	HDL cholesterol
Control	47.21 ± 1.28^b^	52.33 ± 1.14^a^	10.04 ± 0.69^a^	3.63 ± 0.09^a^	33.77 ± 1.26^d^
HFD	66.21 ± 1.65^d^	80.07 ± 1.86^d^	15.68 ± 1.67^c^	33.21 ± 0.12^d^	15.06 ± 1.33^a^
400 mg/kg body weight of extract	43.11 ± 2.01^a^	51.12 ± 2.70^a^	9.76 ± 0.11^a^	2.12 ± 0.24^a^	34.29 ± 0.98^d^
HFD + 100 mg/kg body weight extract	63.24 ± 1.46^d^	76.34 ± 4.11^d^	15.37 ± 0.43^c^	30.04 ± 1.02^d^	16.78 ± 1.54^a^
HFD + 200 mg/kg body weight extract	58.67 ± 0.76^c^	71.35 ± 3.19^c^	14.99 ± 1.04^b^	18.54 ± 0.92^c^	23.11 ± 1.29^b^
HFD + 400 mg/kg body weight extract	50.04 ± 0.48^b^	62.35 ± 2.30^b^	13.77 ± 0.87^b^	11.64 ± 1.03^b^	26.45 ± 0.89^c^
HFD + 400 mg/kg body weight metformin	55.21 ± 0.92^c^	64.76 ± 2.05^b^	13.92 ± 0.92^b^	17.65 ± 1.00^c^	24.68 ± 0.57^b^

Values are mean ± SEM of five determinations and are considered statistically significant at *p* < 0.05. VLDL cholesterol: very-low density lipoprotein cholesterol; HDL cholesterol: high-density lipoprotein cholesterol; LDL cholesterol: low-density lipoprotein cholesterol, HFD; high-fat diet. Concentrations of lipid profile parameters are expressed as mg/dL. Values with different alphabetical superscript are significantly different (*p* < 0.05).

**Table 4:** Specific activities of antioxidant enzymes in the liver of HFD-fed rats following the administration of aqueous extract of *D. cumminsii* leaves to high-fat diet-fed rats.

Group	SOD	Catalase	GSH-Px	GSH-Red	Glucose-6-PD
Control	44.03 ± 2.01^d^	32.03 ± 0.16^e^	78.21 ± 0.97^f^	33.33 ± 1.41^d^	23.71 ± 2.01^d^
HFD	27.54 ± 3.12^a^	14.21 ± 0.32^a^	26.26 ± 1.34^a^	13.18 ± 1.56^a^	9.75 ± 1.98^a^
400 mg/kg body weight of extract	48.76 ± 1.11^e^	38.23 ± 1.23^f^	83.51 ± 1.26	34.10 ± 1.23^d^	24.21 ± 1.65^d^
HFD + 100 mg/kg body weight extract	33.24 ± 1.78^b^	17.65 ± 2.19^b^	42.08 ± 2.10^b^	13.27 ± 1.34^a^	13.45 ± 1.89^b^
HFD + 200 mg/kg body weight extract	37.65 ± 0.99^c^	23.15 ± 0.75^c^	53.15 ± 2.31^c^	17.37 ± 1.39^b^	18.76 ± 3.41^c^
HFD + 400 mg/kg body weight extract	40.19 ± 2.03^c^	27.24 ± 1.14^d^	67.22 ± 1.01^d^	21.23 ± 0.64^c^	7.02 ± 0.26^a^
HFD + 400 mg/kg body weight metformin	41.07 ± 1.02^c^	24.01 ± 1.23^c^	72.92 ± 2.22^e^	20.05 ± 0.68^c^	8.22 ± 0.93^a^

Values are mean ± SEM of five determinations and are considered statistically significant at *p* < 0.05. SOD: superoxide dismutase; GSH-Px: glutathione peroxidase; GSH-Red: glutathione reductase; Glc 6-PD: glucose 6-phosphate dehydrogenase; HFD: high-fat diet. Enzyme activities are expressed as nmol/min/mg protein. Values with different alphabetical superscript are significantly different (*p* < 0.05).

**Table 5:** Levels of malondialdehyde, conjugated dienes, and lipid hydroperoxides in the liver of HFD-fed rats following the administration of aqueous extract of *D. cumminsii* leaves.

Group	Malondialdehyde	Conjugated dienes	Lipid hydroperoxides
Control	3.84 ± 0.37^a^	23.89 ± 2.33^b^	18.07 ± 0.62^b^
HFD	27.21 ± 0.45^d^	75.21 ± 3.07^f^	64.62 ± 3.27^g^
400 mg/kg body weight of extract	3.62 ± 0.54^a^	18.05 ± 0.29^a^	10.12 ± 1.24^a^
HFD + 100 mg/kg body weight extract	16.82 ± 1.79^c^	38.24 ± 0.98^e^	38.76 ± 0.43^f^
HFD + 200 mg/kg body weight extract	12.05 ± 1.06^c^	27.01 ± 1.34^c^	33.21 ± 2.22^e^
HFD + 400 mg/kg body weight extract	7.45 ± 1.92^b^	27.23 ± 1.65^c^	25.32 ± 1.86^c^
HFD + 400 mg/kg body weight metformin	7.53 ± 1.63^b^	30.61 ± 1.53^d^	28.22 ± 0.37^d^

Values are mean ± SEM of ten determinations and are considered statistically significant at *p* < 0.05. HFD: high-fat diet. Conjugated dienes, lipid hydroperoxides, malondialdehyde, and protein carbonyl are expressed as nmol/mg protein. Values with different alphabetical superscript are significantly different (*p* < 0.05).
